# Insulin-like growth factor-I gene therapy reverses morphologic changes and reduces hyperprolactinemia in experimental rat prolactinomas

**DOI:** 10.1186/1476-4598-7-13

**Published:** 2008-01-25

**Authors:** Gloria M Console, Claudia B Herenu, Gisela A Camihort, Georgina C Luna, Maria I Bracamonte, Gustavo R Morel, Rodolfo G Goya

**Affiliations:** 1 Department of Cytology, Histology & Embryology B-CICBA, National University of La Plata, CC455; (1900) La Plata, Argentina; 2 INIBIOLP, Faculty of Medicine, National University of La Plata, CC455; (1900) La Plata, Argentina

## Abstract

**Background:**

The implementation of gene therapy for the treatment of pituitary tumors emerges as a promising complement to surgery and may have distinct advantages over radiotherapy for this type of tumors. Up to now, suicide gene therapy has been the main experimental approach explored to treat experimental pituitary tumors. In the present study we assessed the effectiveness of insulin-like growth factor I (IGF-I) gene therapy for the treatment of estrogen-induced prolactinomas in rats.

**Results:**

Female Sprague Dawley rats were subcutaneously implanted with silastic capsules filled with 17-β estradiol (E_2_) in order to induce pituitary prolactinomas. Blood samples were taken at regular intervals in order to measure serum prolactin (PRL). As expected, serum PRL increased progressively and 23 days after implanting the E_2 _capsules (Experimental day 0), circulating PRL had undergone a 3–4 fold increase. On Experimental day 0 part of the E_2_-implanted animals received a bilateral intrapituitary injection of either an adenoviral vector expressing the gene for rat IGF-I (RAd-IGFI), or a vector (RAd-GFP) expressing the gene for green fluorescent protein (GFP). Seven days post vector injection all animals were sacrificed and their pituitaries morphometrically analyzed to evaluate changes in the lactotroph population. RAd-IGFI but not RAd-GFP, induced a significant fall in serum PRL. Furthermore, RAd-IGFI but not RAd-GFP significantly reversed the increase in lactotroph size (CS) and volume density (VD) induced by E_2 _treatment.

**Conclusion:**

We conclude that IGF-I gene therapy constitutes a potentially useful intervention for the treatment of prolactinomas and that bioactive peptide gene delivery may open novel therapeutic avenues for the treatment of pituitary tumors.

## Background

Pituitary adenomas constitute the most frequent neuroendocrine pathology in humans, comprising up to 15% of primary intracranial tumors [1] and also are the most prevalent pathology in old female rats [2]. In both species, prolactinomas are the most frequent type of pituitary adenoma.

Estrogen exposure has been linked to the formation of prolactinomas in both humans and animals [3-5]. Estrogens, particularly estradiol and diethylstilbestrol, have been shown to induce lactotropic cell tumors within 2–4 weeks in female rats. Women taking oral contraceptives often display increased prolactin (PRL) levels and have increased incidence of prolactinomas, although not all women appear to be equally susceptible to the mitogenic effect of estradiol [6,7].

There are a number of growth factors that are estrogen-dependent and function in lactotropic proliferation, differentiation, and/or transformation. The relatedness of these factors and the significance of each alone are not well-understood. Some of these estrogen-regulated factors are epidermal growth factor (EGF), platelet-derived growth factor (PDGF), transforming growth factor alpha (TGF-α), basic fibroblast growth factor (bFGF), interleukin-2 (IL-2), IL-6, fibroblast growth factor-4 (FGF-4), transforming growth factor-β (TGF-β), insulin-like growth factor-I (IGF-I) and IGFI-II [8,9]. Although estrogen is known to up-regulate IGF-I mRNA in normal rat pituitaries [10], large estrogen-induced pituitary adenomas have been reported to possess decreased levels of IGF-I mRNA. Furthermore, partial remission induced by the anti-estrogen tamoxifen was associated with an increase in the pituitary content of IGF-I mRNA of these adenomas [11]. Also, it has been shown that in primary cultures of rat lactotrophs, estrogen has an antiproliferative action in the presence of insulin or IGF-I [12,13]. In view of this evidence, it was of interest to determine whether *in vivo *overexpression of the gene for rat IGF-I in estrogen-induced rat prolactinomas could be able to restore lacotropic cell morphology and reverse hyperprolactinemia. The present report describes our findings.

## Results

### Effect of IGF-I gene therapy on pituitary lactotroph morphology

**Figure 1:**
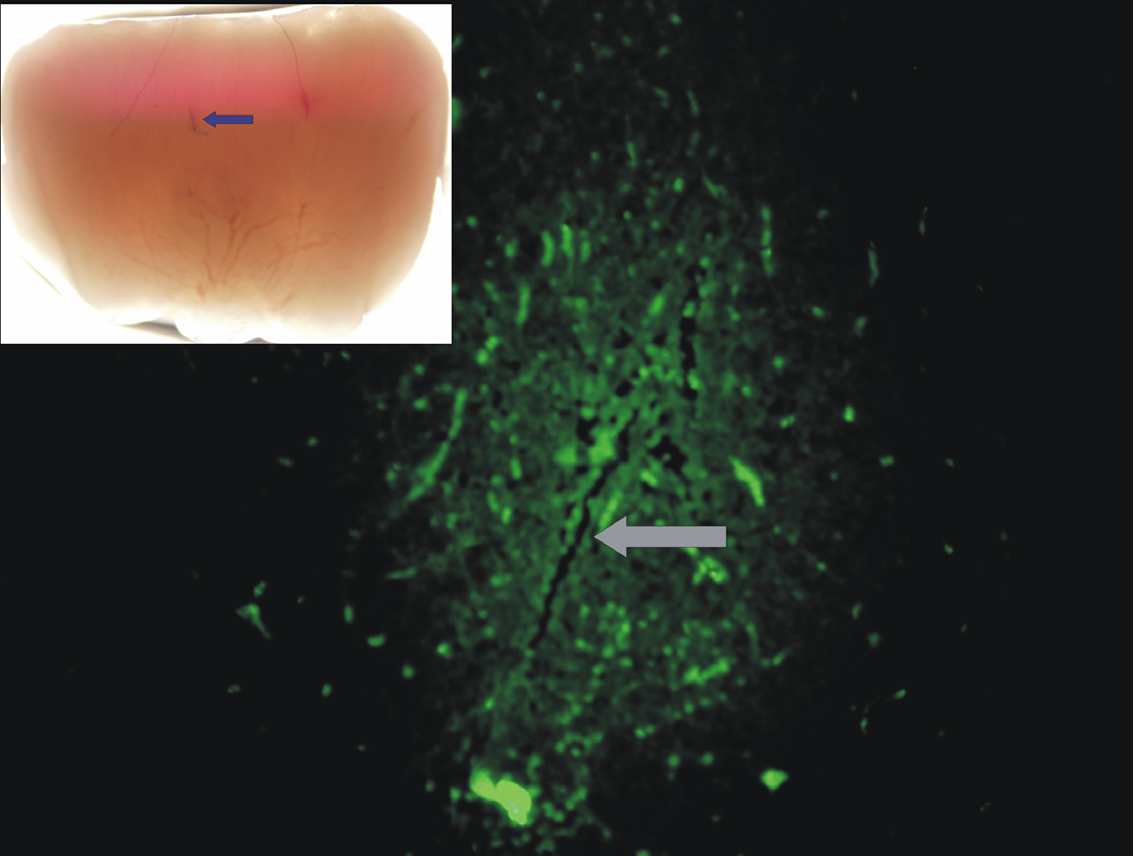
**Expression of transgenic GFP/TK in a pituitary adenoma**. The main panel shows the green fluorescence of transduced cells around the entry point (arrow) of the needle used to stereotaxically deliver RAd-GFP/TK into the tumor Obj. ×20. The **inset **shows a low magnification view of the same pituitary adenoma where an entry point of the needle can be seen (arrow). No structural damage is evident as a consequence of the injection. Obj. ×4.

**Figure 2:**
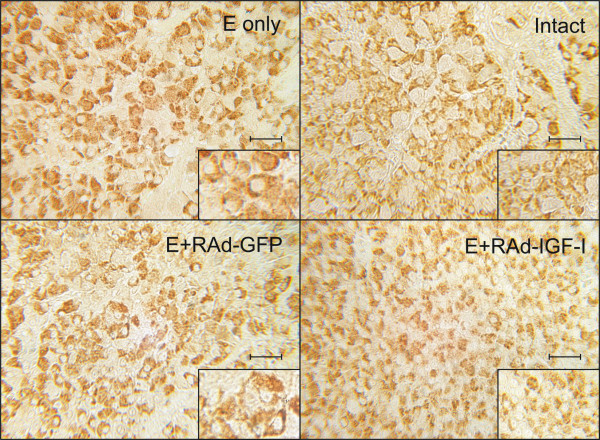
**Effect of IGF-I gene therapy on lactotropic cells of pituitary adenomas**. Representative fields of specifically immunostained PRL-cells in the pituitary gland of an intact (Intact) rat, an animal treated with estrogen for 4 weeks (E only), and animal receiving estrogen for 4 weeks and RAd-GFP during the last experimental week (E + RAdGFP) and an animal receiving estrogen for 4 weeks and RAd-IGF-I during the last experimental week (E + RAd-IGF-I). **Insets**. Higher magnification views of the specimens. EnVision system peroxidase. Bar: 25 μm. Inset: 12.5 μm.

**Figure 3:**
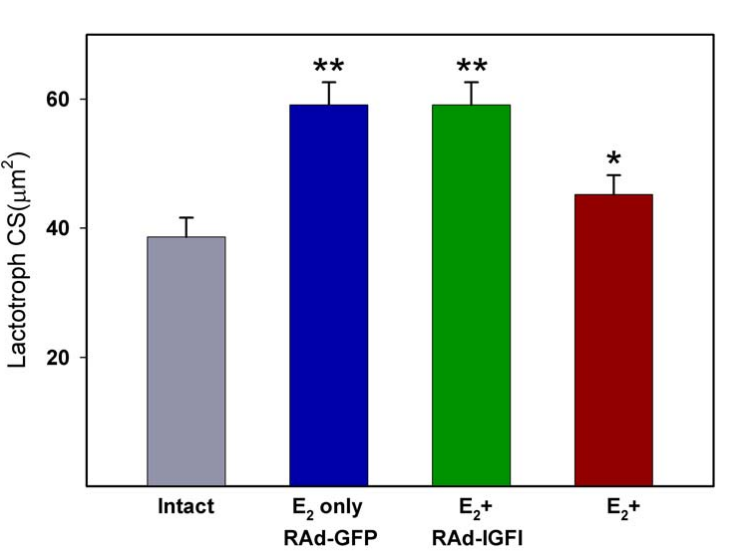
**Effect of IGF-I gene therapy on lactotropic cell size**. Lactotroph cell surface (CS) in the pituitaries of animals submitted to no treatment (intact), control treatments (E_2 _only and E_2 _+ RAdGFP) or IGF-I gene therapy (E_2 _+ RAd-IGF-I). Columns represent mean values whereas bars over columns represent SEM values. Five pituitaries per group were assessed. Asterisks indicate a highly significant difference from the intact group.

**Figure 4:**
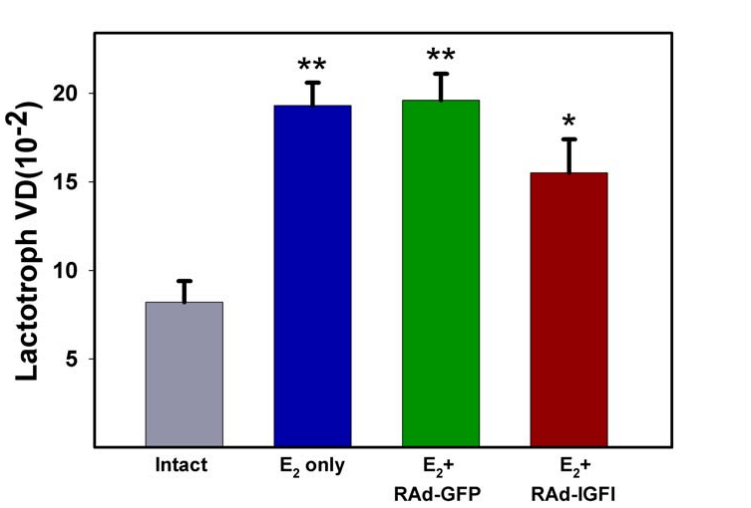
**Effect of IGF-I gene therapy on lactotropic volume density (VD)**. Data correspond to the pituitaries of the same animals shown in Fig 3. Details are as in Fig. 3.

Stereotaxic injection of RAd-GFP (an adenoviral vector expressing a chimeric variant of green fluorescent protein; see Methods) into pituitary adenomas induced a significant expression of GFP around the track of the needle (Fig. 1) without significantly damaging the gland (Fig. 1, inset). As expected, three weeks of estrogen treatment induced a significant increase in pituitary size (data not shown) as well as clear changes in the lactotropic cell population, already evident when pituitary sections were qualitatively assessed (Fig 2). Estrogen-induced pituitary adenomas showed a significant (p < 0.01) increase in lactotropic cell size as compared with their intact counterparts (Fig. 3; also see Fig. 2). Seven days of IGF-I gene therapy significantly reversed this change (p < 0.01) although lactotroph cell surface (CS) was still higher than in intact animals (Fig. 3; also see Fig. 2). Lactotroph volume density (VD) was higher in estrogen-induced pituitary adenomas when compared with the pituitaries of intact animals (Fig. 4). Again, IGF-I gene therapy significantly (p < 0.05) reversed this alteration although lactotroph VD in RAd-IGF-I injected adenomas remained higher than in normal intact glands (Fig. 4).

### Effect of intrapituitary tumor IGF-I gene therapy on serum PRL levels

**Figure 5:**
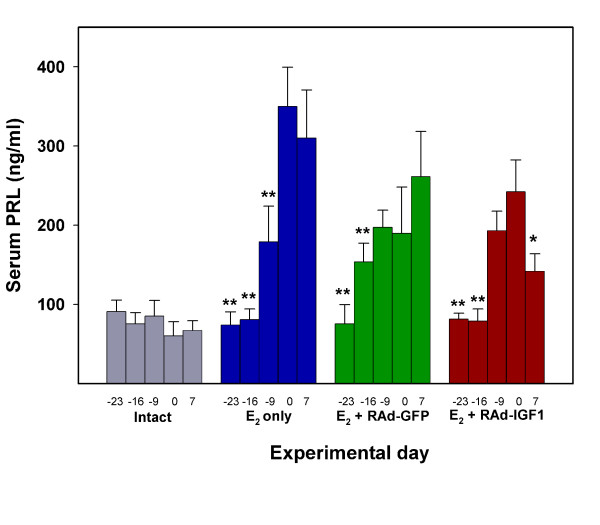
**Effect of IGF-I gene therapy on serum PRL levels**. Serum PRL was measured in blood samples serially taken from Experimental day -23 to Experimental day 7. The number of rats used was 5 for all groups except for group E_2 _+ RAd-IGF-I where 7 animals were assessed Asterisks indicate significant differences from serum PRL on experimental day 0 (vector injection); *: P < 0.05; **: P < 0.01. Other details are as in Fig. 3

As expected, estrogen administration induced a marked hyperprolactinemia in the animals. Seven days after stereotaxic intrapituitary injection of RAd-IGF-I but not RAd-GFP, a significant fall in serum PRL occurred in the estrogen-treated rats (Fig. 5). Nevertheless, serum PRL of the RAd-IGF-I-treated rats remained higher than in the intact rats.

## Discussion

Current therapies for pituitary tumors include surgery and radiotherapy, as well as pharmacological approaches for some types and, although important advances have been made in the treatment of pituitary tumors with these strategies, a fully satisfactory therapy is not yet available [14]. In this context, gene therapy appears as a potentially useful alternative for the treatment of pituitary tumors.

Early studies showed that a herpes simplex virus type-1 (HSV1)-derived vector was highly effective *in vivo *for gene transfer in rat pituitary prolactinomas [15]. An adenoviral vector, RadTK, harboring the HSV-1 thymidine kinase (TK) suicide gene under the control of the human cytomegalovirus (hCMV) promoter, was used to transfer the TK gene to GH_3 _and AtT_20 _rodent pituitary tumor cells. Incubation of RadTK-treated GH_3 _and AtT_20 _cells with the prodrug ganciclovir (which after phosphorylation by viral TK becomes toxic) caused ample destruction of the cultures [16]. In the same study, estrogen/sulpiride-induced rat prolactinomas were stereotaxically injected with the same RadTK. Subsequent injection of the host animals with two daily intrapituitary doses of 25 mg ganciclovir/kg for 7 days succeeded in partially reducing tumor size and serum PRL levels. Another type of gene therapy strategy for the treatment of pituitary cancer is that based on the transfer of a gene(s) with the ability to rescue the normal phenotype of tumor cells. This approach has been implemented in mice heterozygous for the retinoblastoma (RB) tumor suppressor gene (*Rb*^+/- ^mice) which develop and succumb to characteristic pituitary intermediate lobe melanotroph tumors [17]. Intracranial delivery of an adenoviral vector harboring the human RB cDNA to mice carrying actively growing melanotrophic tumors significantly reduced tumor growth and prolonged animal survival [18].

The present study is the first to implement effective pituitary tumor gene therapy using the gene for a bioactive peptide. Within the time frame used, the treatment seems to act mainly on PRL cell size and secretory activity rather than on PRL cell density (CD, data not shown). Our results are consistent with the above mentioned reports documenting that large estrogen-induced pituitary adenomas possess decreased pituitary levels of IGF-I mRNA and that the anti-estrogen tamoxifen increased the IGF-I mRNA content in the involuting adenomas [11]. Our results are also consistent with the evidence that in rat lactotrophs, estrogen has an antiproliferative action in the presence of insulin or IGF-I [12,13]. Furthermore, our data showing that IGF-I gene therapy reduces PRL secretion in estrogen-induced prolactinomas are in line with an early study in rat pituitary cells reporting that IGF-I inhibited the estrogen induced rise in PRL and lactotroph pituitary transcription factor-1 (Pit-1) mRNA levels in females. This study also documented that the inhibitory effect of IGF-I on estrogen-induced PRL and lactotroph Pit-1 mRNA levels did not occur in male pituitary cells [19].

The mechanism by which pituitary overexpression of IGF-I reduces lactotropic cell size and inhibits PRL secretion is not clear. Although estrogen is known to increase pituitary expression of IGF-I mRNA, it also increases the expression of IGF binding protein 2 (IGF-BP2) mRNA [10]. Since our approach involves pituitary overexpression of transgenic IGF-I, probably not paralleled by a concomitant increase in IGF-BP2, the possibility exists that high levels of free IGF-I exert an inhibitory action on the lactotrophs. It is well-established that IGF-I exerts a potent negative feedback on somatotropic cells [20-22]. Since lactotrophs and somatotrophs have a common origin and share a number of physiologic features, it seems conceivable that high pituitary concentrations of free IGF-I could have an inhibitory action on lactotropic cells. Clearly, further work is necessary to clarify this matter.

We conclude that IGF-I gene therapy constitutes a potentially useful intervention for the treatment of prolactinomas and that bioactive peptide gene delivery may open novel therapeutic avenues for the treatment of pituitary tumors.

## Methods

### Adenoviral vectors

#### RAd-IGFI

**Figure 6:**
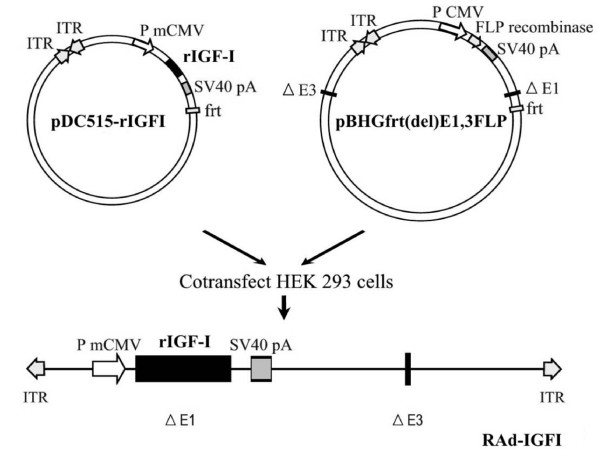
**Diagrammatic representation of the procedure used to construct RAd-IGF-I**. An expression cassette containing the rat IGF-I cDNA (form B) flanked by the mCMV promoter (PmCMV) and the SV40 polyadenylation signal (SV40pA), upstream and downstream, respectively, was inserted in the shuttle vector pDC515. Subsequently, pDC515 and the genomic plasmid pBHGfrt(del)E1,3 FLP (which consists of the entire genome of adenovirus 5 (Ad 5), containing deletions in the regions E1 and E3) were cotransfected in permissive HEK 293 cells. Enzyme-directed recombination occurred in cotransfected cells, giving rise to RAd-IGFI. frt, recognition element for the yeast FLP recombinase; ITR, inverted terminal repeats; Δ1 and Δ3: deletions in the Ad 5 genome.

A recombinant adenoviral (RAd) vector harboring the rat IGF-I gene (kindly donated by Dr. Peter Rotwein, Oregon Health Sciences University) was constructed in our laboratory by a variant of the two plasmid method employing the AdMax^® ^plasmid kit (Microbix, Ontario, Canada) [23]. Briefly, the cDNA coding for rat IGF-I gene (obtained from the mRNA for the IGF Ib precursor form [24]) was excised from plasmid pBluescript KS, subcloned in pCA14 and inserted in the multiple cloning site (MCS) of shuttle pDC515 which contains an expression cassette consisting of the mouse cytomegalovirus promoter (mCMV) and the simian virus 40 (SV40) polyadenylation signal, immediately upstream and downstream to the MCS, respectively. The second plasmid of the kit, the genomic plasmid pBHGfrt(del)E1,3 FLP, consists of the entire genome of adenovirus 5 (Ad5), containing deletions in the regions E1 and E3. In cotransfected HEK293 cells, FLP recombinase is readily expressed and efficiently catalyzes the site-directed recombination of the expression cassette of pDC515 into pBHGfrt(del)E1,3 FLP, thus generating the genome of the desired recombinant adenoviral vector, RAd-IGF-I (Fig. 6). The newly generated RAd was rescued from HEK293 cell lysates and plaque purified. It was further purified by ultracentrifugation in CsCl gradient. Final virus stocks were titrated by a serial dilution plaque assay.

#### RAd-(GFP/TK)fus

An adenoviral vector termed RAd-(GFP/TK)fus, or RAd-GFP for short, was constructed in our laboratory following the general procedures outlined above and was used as a control vector in the gene therapy studies. The vector harbors a hybrid gene encoding the Aequorea victoria enhanced green fluorescent protein fused to herpes simplex virus type 1 thymidine kinase (GFP/TK)fus (a kind gift from Dr. Jacques Galipeau, McGill University, Montreal, Canada). This hybrid gene is driven by the mouse CMV promoter. The vector was expanded in 293 cells and purified and titrated as indicated above.

### Animals

Young female Sprague-Dawley rats were housed in a temperature-controlled room (22 ± 2°C) on a 12:12 h light/dark cycle. Food and water were available ad libitum. All experiments with animals were done following the Animal Welfare Guidelines of NIH (INIBIOLP's Animal Welfare Assurance No A5647-01).

### Experimental design for *in vivo *IGF-I gene therapy

At experimental day -23 all animals but those of the intact group, were subcutaneously implanted a silastic capsule filled with 17-β estradiol. Small blood samples (0.4 ml) were taken from the tail veins of all rats on experimental day -16, -9, 0, day +2 and day +7 for PRL assay. On experimental day 0, some of the rats received bilateral 1.5 μl intrapituitary injections containing 3 × 10^9 ^plaque forming units (pfu) of either RAd-GFP or RAd-IGF-I, respectively. For this purpose, rats were anesthetized with injection of ketamine hydrochloride (40 mg/kg, i.p.) and xylazine (8 mg/kg, i.m.), and placed in a stereotaxic frame. In order to access the pituitary region, the tip of a 26 gauge needle fitted to a 10 μl syringe was brought to the following coordinates relative to the bregma: 5.5 mm posterior, 9.6 mm ventral and 0.7 mm right and left [25]. One week after intrapituitary injection rats were killed by decapitation. Pituitaries were immediately dissected, fixed and processed for routine histological studies.

### Immunohistochemistry

Stated in brief, pituitary tissues from 5 animals of each group were fixed in Bouin's fluid and embedded in paraffin. Serial sections of 4 μm were obtained at different levels of the blocks following a ventral-to-dorsal sequence. The sections were immunostained, and then incubated for 1 h at room temperature with the anti-PRL primary antiserum (murine, Dako, CA, USA), diluted 1:100. Thoroughly washed sections were then treated for 30 min with a ready-to-use EnVision reaction system (Dako, CA, USA). The peroxide-sensitive chromogen was diaminobenzidine. In all instances, the specificity of the primary antiserum was monitored either by observing its ability to block the immunocytochemical reaction after its preabsorption with an excess of the related antigen or by its replacement with normal rabbit serum or phosphate-buffered saline [26].

### Image analysis

Morphometry was performed as reported in detail previously [27]. Measurements of pituitary cells were made by means of an image-analysis system (Imaging Technology, Optimas 5.2). The immunostained lactotrope cells and the reference area (RA) were analyzed in each field on an average of ten micrographs taken from two levels (e.g. **a **and **b**) in the groups studied. These measurements were recorded and processed automatically and the following parameters subsequently calculated: volume density (VD = Σ cell area/RA) and cell size (CS) (the mean of individual cell area, expressed in μm2). Reference area (RA) represents the adenohypophyseal (*pars distalis*) area scanned, in which pituitary PRL cells were scored. Then, with the sum (Σ) of the individual areas (A), referred to as RA, we obtained volume density (VD), which indicates cell mass according to a generally accepted concept.

### Hormone assays

Serum levels of PRL were measured by a specific radioimmunoassay using the rat materials provided by Dr. A. F. Parlow, Pituitary Hormones and Antisera Center, UCLA Med. Center, U.S.A. Iodination grade hormones were radiolabeled by the Iodo-Gen^® ^method and purified on PD-10 Sephadex^® ^G-25 M columns (Pharmacia, Uppsala, Sweden) equilibrated with 0.01 M phosphosaline, pH 7.6. A 1/10 goat anti-rabbit IgG in 0.9% NaCl was used to separate bound from free hormone. Serum concentrations of PRL were expressed in terms of NHPP rPRL RP-3.

### Statistical analysis

The one way analysis of variance (ANOVA) were used to evaluate group differences. Tukey's method was chosen as a post hoc test. In Figs. 3 and 4 pair comparisons were made between intact and each of the other experimental groups. Also, RAd-GFP vs. RAd-IGF1 pairs were compared in both figures. In Fig. 5 one-way ANOVA was applied to each set of data (intact, E2, etc) and pair comparisons were made between Exptl. Day 0 and each of the other time points.

## Authors' contributions

GMC supervised the morphometric analysis of the pituitaries and made a major contribution to the preparation of the manuscript; CBH constructed the viral vectors used and performed the surgical procedures; GAC and GML executed the different aspects of the morphometric analysis of pituitaries; MIB carried out the histological procedures involved in this study; GRM performed the studies involving immunofluorescence analysis; RGG conceived of the study and participated in its design and coordination as well as in writing the manuscript. All authors read and approved the final manuscript.
